# Video-Rate Bioluminescence Imaging of Matrix Metalloproteinase-2 Secreted from a Migrating Cell

**DOI:** 10.1371/journal.pone.0025243

**Published:** 2011-09-28

**Authors:** Takahiro Suzuki, Chihiro Kondo, Takao Kanamori, Satoshi Inouye

**Affiliations:** 1 Department of Biochemistry, School of Dentistry, Aichi-Gakuin University, Nagoya, Japan; 2 Yokohama Research Center, JNC Corporation, Yokohama, Japan; Stanford University, United States of America

## Abstract

**Background:**

Matrix metalloproteinase-2 (MMP-2) plays an important role in cancer progression and metastasis. MMP-2 is secreted as a pro-enzyme, which is activated by the membrane-bound proteins, and the polarized distribution of secretory and the membrane-associated MMP-2 has been investigated. However, the real-time visualizations of both MMP-2 secretion from the front edge of a migration cell and its distribution on the cell surface have not been reported.

**Methodology/Principal Findings:**

The method of video-rate bioluminescence imaging was applied to visualize exocytosis of MMP-2 from a living cell using *Gaussia* luciferase (GLase) as a reporter. The luminescence signals of GLase were detected by a high speed electron-multiplying charge-coupled device camera (EM-CCD camera) with a time resolution within 500 ms per image. The fusion protein of MMP-2 to GLase was expressed in a HeLa cell and exocytosis of MMP-2 was detected in a few seconds along the leading edge of a migrating HeLa cell. The membrane-associated MMP-2 was observed at the specific sites on the bottom side of the cells, suggesting that the sites of MMP-2 secretion are different from that of MMP-2 binding.

**Conclusions:**

We were the first to successfully demonstrate secretory dynamics of MMP-2 and the specific sites for polarized distribution of MMP-2 on the cell surface. The video-rate bioluminescence imaging using GLase is a useful method to investigate distribution and dynamics of secreted proteins on the whole surface of polarized cells in real time.

## Introduction

Matrix metalloproteinases degrade extracellular matrix proteins and regulate cell adhesion and migration. The polarized distribution of these proteinases has been demonstrated in migrating cells [1]–[5]. MMP-2 is one of the enzymes in degradation of basement membrane collagen and has a major role in cancer cell invasion. Regulatory mechanisms and inhibitors on MMP-2 protease activity have been extensively studied in cancer research [5]–[9]. Up-regulations of gene expression and secretion of MMP-2 in both cancer cells and surrounding stromal cells have been shown to promote cancer progression and metastasis [7]. In addition, MMP-2 plays important roles in immune and neural cells under physiological and pathological conditions [5], [7], [9]–[11].

On the cell surface, the inactive form of MMP-2 (pro-MMP-2) binds to tissue inhibitor of metalloproteinase-2 (TIMP-2) [12], which associated with the membrane type 1-matrix metalloproteinase (MT1-MMP; also called MMP-14) [13], and then the amino terminal peptide of pro-MMP-2 is cleaved by MT1-MMP to give intermediate form [14]. The intermediate form binds to integrin αvβ3 at the cell surface, and full active MMP-2 is produced [15]–[17]. The polarized localization of MMP-2 on lamellipodia and invadopodia of a cell [1]–[4] were shown by the immunohistochemical studies using a fluorescence-labeled antibody, and the activated MMP-2 is considered to be localized in front of a migrating cell with protease activity. However, the regulatory mechanism of MMP-2 secretion is still poorly understood, and exocytotic secretion of MMP-2 from the migrating cells has not been visualized in real time.

**Figure 1:**
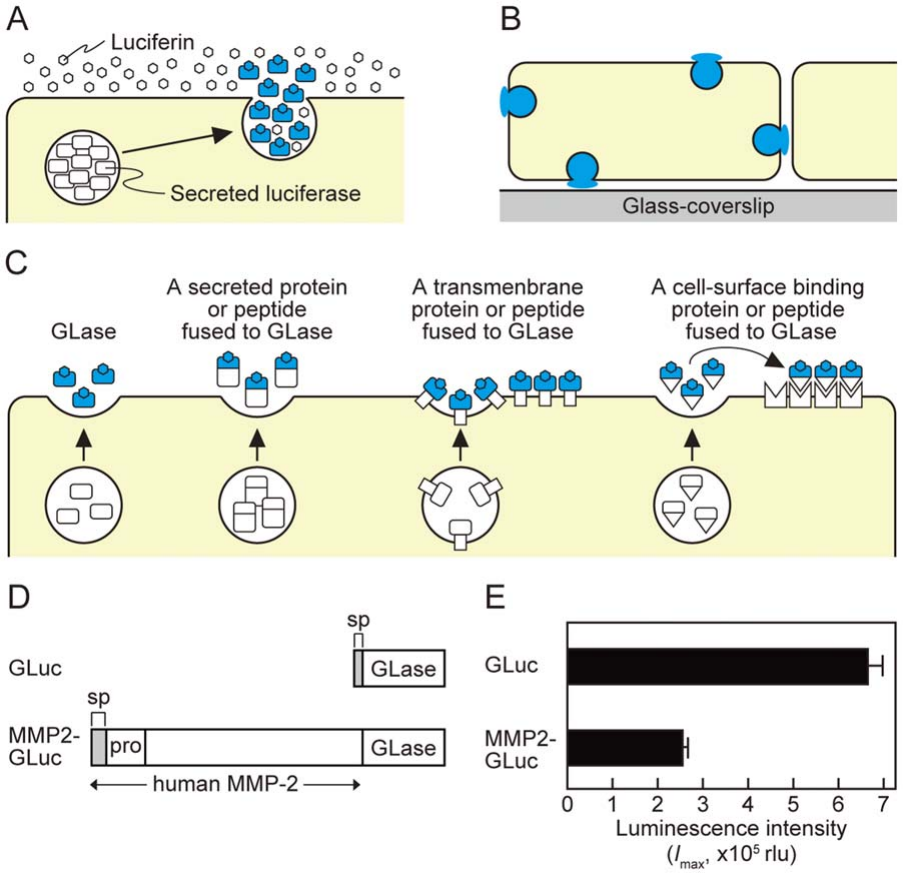
Bioluminescence imaging of GLase as a reporter protein to visualize proteins on the surface of mammalian cells. (A) Schematic representation of imaging principles by GLase bioluminescence. (B) Detectable area of exocytosis in a single mammalian cell using GLase bioluminescence imaging method. (C) Schematic representation of the secretion of GLase and the fusion protein of GLase from mammalian cells and its binding on the cell surface. (D) Schematic representation of expression vectors for GLase and MMP2-GLase. GLuc (pcDNA3-GLuc); GLase with the signal peptide sequence, MMP2-GLuc (pcDNA3-hMMP2-GLuc); human MMP2 preproprotein fused to GLase. (E) GLase activity in the conditioned medium of HeLa cells. HeLa cells were transfected with pcDNA3-GLuc or pcDNA3-hMMP2-GLuc and cultured for 24 hr followed by incubation with HBSS for 60 min at 37°C. The luminescence activity in the conditioned medium was determined using a luminometer.

To visualize an individual exocytotic event in a single living cell, total internal reflection fluorescence (TIRF) imaging has been mainly applied and can only visualize within the evanescent field [18]–[22]. For example, exocytosis of secretory vesicles possessing the fusion protein of low-density lipoprotein receptor with green fluorescence protein was polarized toward the leading edge in migrating fibroblasts [21]. However, some exocytotic events around the leading edge of cell, especially within 1∼2 µm from the cell edge, could not be detected clearly by TIRF imaging, because lamellipodia in a migrating cell are often wavering and are detached from the cover slip [21]. This limitation of fluorescence imaging for protein secretion prompted us to apply video-rate bioluminescence imaging for the whole surface of a cell [23]–[26] (Figure 1A and B).

Here, we introduced an electron multiplying charge-coupled device (EM-CCD) camera as a sensitive detector and established the method of a video-rate bioluminescence imaging with the combination of EM-CCD camera and the secretory luciferase, *Gaussia* luciferase (GLase). As a result, this method allowed us to visualize exocytotic protein secretion with a time resolution of 30–500 ms per image, and was applied to investigate the secretory dynamics of MMP-2 in a migrating HeLa cell. We successfully demonstrated the specific sites for both secretion and binding of MMP-2 on the cell surface.

## Results and Discussion

### Video-rate bioluminescence imaging of protein secretion from a single HeLa cell with an EM-CCD camera and *Gaussia* luciferase

**Figure 2:**
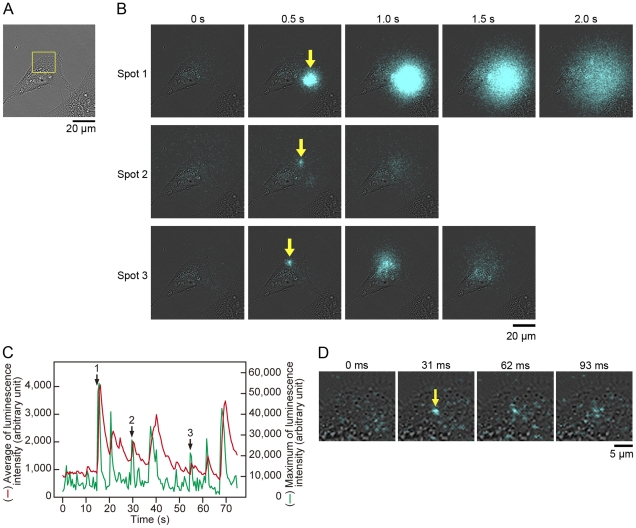
Video-rate bioluminescence imaging of GLase secretion from a single HeLa cell using an EM-CCD camera. HeLa cells were transfected with pcDNA3-GLuc and cultured for 24 h. (A) Bright-field image of a single cell. Objective lens; 40×. (B) Bioluminescence image of GLase secretion obtained from all area of bright-field image (A). Luminescence signals were recorded with an exposure time of 500 ms per image for 75 s (Video image is in Video S1). Yellow arrows in the images at 0.5 s indicate the luminescence spots in three spots 1–3. (C) Time-dependent changes of the average (red line) and maximum (green line) of luminescence intensities from GLase secretion. The arrows with numbers of 1–3 indicate the luminescence positions, shown as yellow arrows at 0.5 s in (B). (D) Magnified luminescence image of GLase secretion obtained from the area of yellow square in (A). Luminescence signals were acquired with an exposure time of 30.5 ms per image for 4.7 s (Video image is in Video S3).

Previously, we visualized the exocytotic events of protein secretion and the protein targeting on the cell surface of CHO-K1 and PC12D cells using GLase [26]. The secreted GLase is the smallest luciferase (168 amino acid residues) and it is useful reporter protein for bioluminescence imaging over the cells (Figure 1C), but we could not distinguish between the secretory protein by exocytosis and the localized protein on the cell surface in real time due to low luminescence sensitivity of a photon counting camera and a CCD camera [26]. Prior to the visualization of MMP-2 secretion from a migrating HeLa cell, we established an improved bioluminescence video imaging system using a high-speed sensitive EM-CCD camera as a detector attached to a microscope. The EM-CCD camera showed very high quantum efficiency with over 90% and the luminescence video images were recorded at full spatial resolution allowing acquisition of 32 frames per second (with a minimum exposure time of 30.5 ms per image) on a hard disk. The performance of our improved imaging system with EM-CCD camera was evaluated using GLase secretion from HeLa cells. The cells transiently expressing GLase were cultured on a glass-bottom dish and were incubated with Hank's Buffered Salt Solution (HBSS) containing luciferin (coelenterazine). With an exposure time of 30.5, 100, or 500 ms per image, the luminescence video images of GLase secretion from a single HeLa cell with a high numerical aperture 40× objective lens (NA 1.30) were recorded (Figure 2; Videos S1, S2, and S3). The luminescence video images with an exposure time of 500 ms per image clearly showed that GLase was released from specific sites on a HeLa cell and rapidly diffused to the outside of the cell (Figure 2B; Video S1). The luminescence intensities in each image could be used as an indicator for the amounts of secreted protein and the number of exocytotic sites. Thus, we could estimate time-dependent changes of GLase secretion from a single cell. The estimated values of luminescence intensities on the video image showed that the amounts of GLase secreted from a cell fluctuated in a short time and the secretion of GLase varied in different sites on the cell (Figure 2C; Video S1). Luminescence video images with the shorter exposure times of 30.5 and 100 ms showed smaller luminescence spots could be observed and these small spots might be individual exocytotic events of GLase (Figure 2D; Video S2 and S3).

In our previous imaging system using a photon-counting camera with a long exposure time for 1 min, the intercellular weak luminescence signals were detected after 10 min by incubating with coelenterazine [26]. This result suggested that coelenterazine was slowly incorporated into a cell by endocytosis and this weak luminescence signals were explained by the luminescence reaction of GLase with coelenterazine. On the other hand, the present imaging study using a high-speed sensitive EM-CCD camera was performed with shorter exposure times within 500 ms and the luminescence images of secreted GLase were obtained within 15 min. In our imaging conditions, the diffusive luminescence signals of secreted GLase from a HeLa cell were observed without any significant increase in background luminescence signals in the cells (Figure 2B and D, and Videos S1, S2, and S3). Thus, the time resolution and the detection sensitivity of luminescence signals were significantly improved, accompanying high signal-to-noise ratio of luminescence signals of secreted GLase for detection.

### Expression and characterization of the fused protein of MMP-2 to GLase in HeLa cells

**Figure 3:**
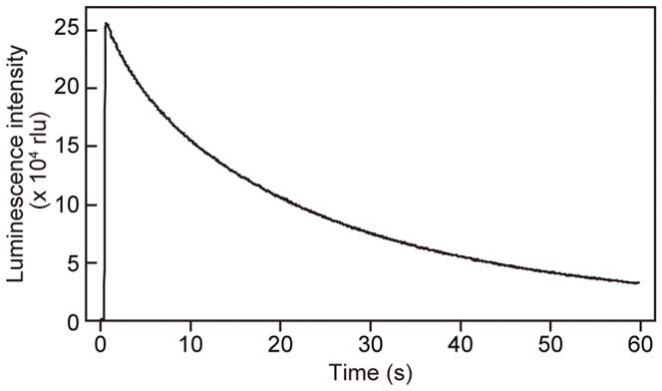
Time-dependent decrease of luminescence intensity of MMP2-GLase with coelenterazine. Luminescence intensity of MMP2-GLase with coelenterazine (3 µg/ml) is recorded for 60 s. HeLa cells were transfected with pcDNA3-hMMP2-GLuc and cultured for 24 h. After removing the medium, the cells were incubated with HBSS for 60 min at 37°C and the luminescence activity in HBSS was determined using a luminometer, as described in *Materials and Methods*.

To express the fusion protein of MMP-2 with GLase in HeLa cells, the expression vector of MMP2-GLase (Figure 1D) was constructed and was transfected into HeLa cells, which is known to express endogenous MMP-2 [27], [28]. The luminescence activity of MMP2-GLase secreted into the conditioned medium was determined with a luminometer and the luminescence intensity of MMP2-GLase for bioluminescence video imaging analysis was observed (Figure 1E). The luminescence intensity of MMP2-GLase was decreased in a time-dependent manner (Figure 3) as previously reported [26], [29], [30].

**Figure 4:**
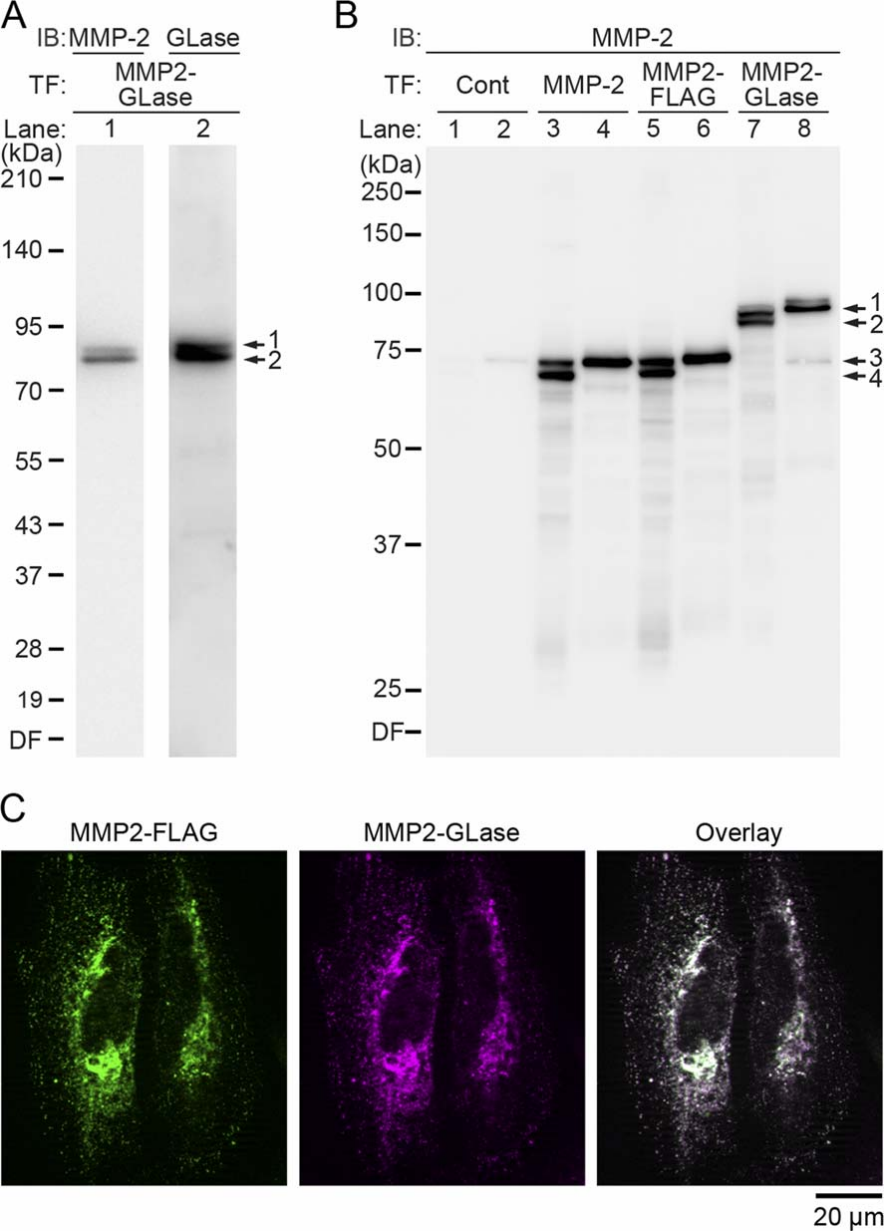
Characterization of MMP2-GLase expressed in HeLa cells with immunochemical analyses. MMP2-GLase expressed in HeLa cells was identified by Western blot (A and B) and immunofluorescence (C) analyses. (A) Western blot analysis of MMP2-GLase in cell lysate using anti-MMP-2 and anti-GLase antibodies (MMP-2 and GLase for lane 1 and lane 2, respectively). HeLa cells were transfected with pcDNA3-hMMP2-GLuc for the expression of MMP2-GLase (MMP2-GLase) and cultured for 24 h, and proteins in the cell lysate were separated in a 5–20% gel. The numbers on the left margin represent the molecular weight of size maker proteins (XL ladder, Promega). The molecular sizes of two bands by arrow 1 and 2 correspond to the pro-form (89.4 kDa) and processed form (80.3 kDa) of MMP2-GLase, respectively. (B) Western blot analyses of the cell lysate and the conditioned medium expressing MMP2-GLase, MMP2-FLAG, and wild type MMP-2 using anti-MMP-2 antibody (MMP-2). HeLa cells transfected with pcDNA3 (as a control vector: lane 1 and 2), pcDNA3-hMMP2 (lane 3 and 4), pcDNA3-hMMP2-Flag (lane 5 and 6), or pcDNA3-hMMP2-GLuc (lane 7 and 8) (Cont, MMP-2, MMP2-FLAG, and MMP2-GLase, respectively) were cultured for 24 h, and further cultured for 24 h in HBSS. Proteins in the cell lysate (lane 1, 3, 5, 7) and the concentrated proteins from the conditioned medium of HBSS (lane 2, 4, 6, 8) were separated in a 10% gel. The numbers on the left margin represent the molecular weight of size maker proteins (Precision Plus Protein All Blue standards, Bio-Rad). Two bands indicated by arrow 1 and 2 at the right margin correspond to pro- and processed forms of MMP2-GLase, respectively. Two bands indicated by arrow 3 and 4 at the right margin correspond to pro- and processed forms of MMP-2, respectively. Endogenous pro- and processed MMP-2 (71.0 kDa and 62.0 kDa) slightly detected in lane 1 and 2, exogenous pro- and processed MMP-2 in lane 3 and 4, and pro- and processed MMP2-FLAG (72.2 kDa and 63.2 kDa) in lane 5 and 6. (C) Immunofluorescence images of MMP2-FLAG (Green) and MMP2-GLase (Magenta) co-expressed in HeLa cells. Overlay; Merged image of the both fluorescence images. IB, immunoblot; TF, transfection; DF, dye front.

MMP2-GLase expressed in HeLa cells was identified by Western blot analyses using anti-MMP-2 and anti-GLase antibodies (Figure 4A and B). On SDS-PAGE analysis under reducing conditions, pro- and processed forms of MMP2-GLase were detected in the cell lysate with both antibodies (Figure 4A), indicating that MMP2-GLase expressed and processed correctly. In the conditioned medium, pro-form of MMP2-GLase was only detected as well as exogenous MMP-2 and MMP2-FLAG (Figure 4B lane 4, 6 and 8). These results suggested that MMP2-GLase, as well as exogenous MMP-2 and MMP2-FLAG, was secreted as pro-form and then a processed form was bound on cell surface. Further, the co-localization of MMP2-GLase with MMP2-FLAG on the cell surface was confirmed by immunofluorescence analysis using anti-GLase antibody and anti-Flag antibody. As a result, MMP2-GLase was co-localized with MMP2-FLAG (Figure 2C), supporting that MMP2-GLase expressed in HeLa cells was secreted as pro-form, and was processed for activation on the cell surface.

### Identification of both secretory and membrane-associated forms of MMP-2 on the cell surface in a migrating HeLa cell by video-rate bioluminescence imaging

**Figure 5:**
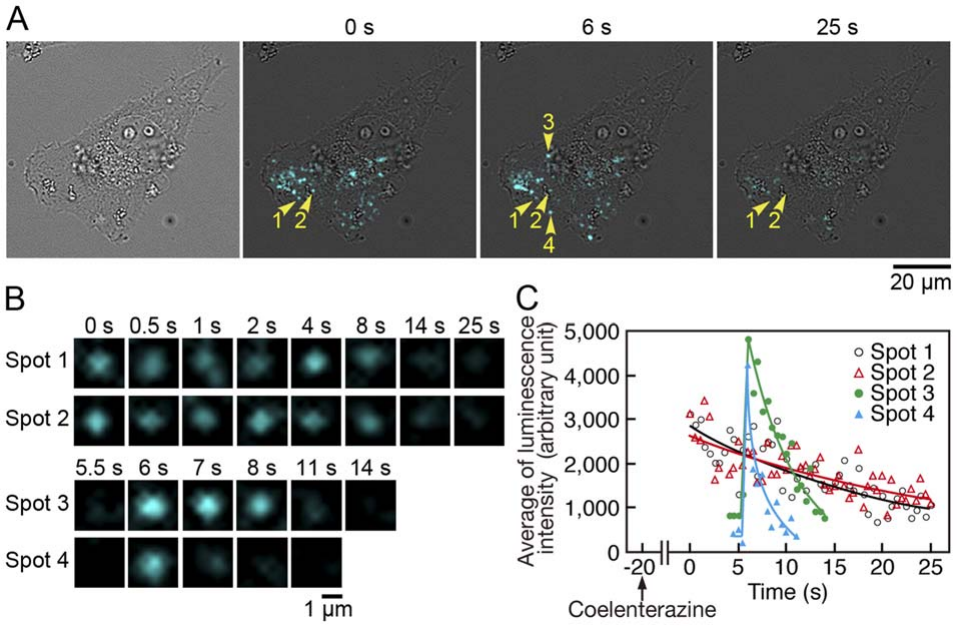
Identification of MMP2-GLase being secreted and bound on the cell surface of a HeLa cell using bioluminescence imaging. Bioluminescence imaging of HeLa cells transiently expressing MMP2-GLase. Objective lens; 40×. Luminescence signals recording was started after 20 s from the addition of HBSS buffer containing coelenterazine with an exposure time of 500 ms for 75 s (Video image is in Video S4). (A) The bright-field image (the left panel), and luminescence images acquire at 0, 6, and 25 s. Two continuous luminescence spots (spot 1 and 2) are indicated by yellow arrowheads labeled with 1 and 2, respectively, and two transient diffusive luminescence spots (spot 3 and 4) are indicated by yellow arrowheads labeled with 3 and 4, respectively. (B) Magnified luminescence images of spot 1–4 indicated by arrowheads labeled with 1–4 in (A). (C) Time-dependent changes of the average luminescence intensity of spot 1–4 indicated by arrowheads labeled with 1–4 in (A).

To visualize the secretion and localization of MMP-2 on the cell surface, video-rate bioluminescence imaging of MMP2-GLase expressed in HeLa cells was performed using the improved imaging system with an EM-CCD camera. With an exposure time of 500 ms per image, the luminescence video image of a migrating HeLa cell was obtained using the 40× objective lens. The transient luminescence spots and continuous luminescence spots were detected on the cell (Figure 5A; Video S4). The continuous luminescence spots within 3 µm in diameter retained and gradually disappeared in 1 min after adding of coelenterazine (Figure 5B and C). This slow decrease of the continuous luminescence signals seemed to be time-dependent decrease of luminescence activity of MMP2-GLase (Figure 3). The results of Western blot analyses suggested that the pro-form and processed form of MMP2-GLase could bind on the cell surface (Figure 4B), and the video images supported that the continuous luminescence spots represented the signals of MMP2-GLase binding on the cell surface. On the other hand, the transient luminescence spots could be interpreted as MMP2-GLase being secreted from the cell. The individual luminescence signals for secreted GLase were rapidly diffused from cells (Figure 2B and video S1) and the luminescence signals of MMP2-GLase were remained as spots for a few seconds on the cells (spot 3 and 4 in Figure 5B, and also see transient luminescence signals in Video S4). These results indicated that MMP2-GLase could be used as a reporter for MMP-2 secretion. Thus, we could visualize two distinct types of MMP-2, secreted as well as membrane-associated MMP-2, on the cell surface in real time.

### Characterization of exocytotic MMP-2 secretion in a migrating HeLa cell

**Figure 6:**
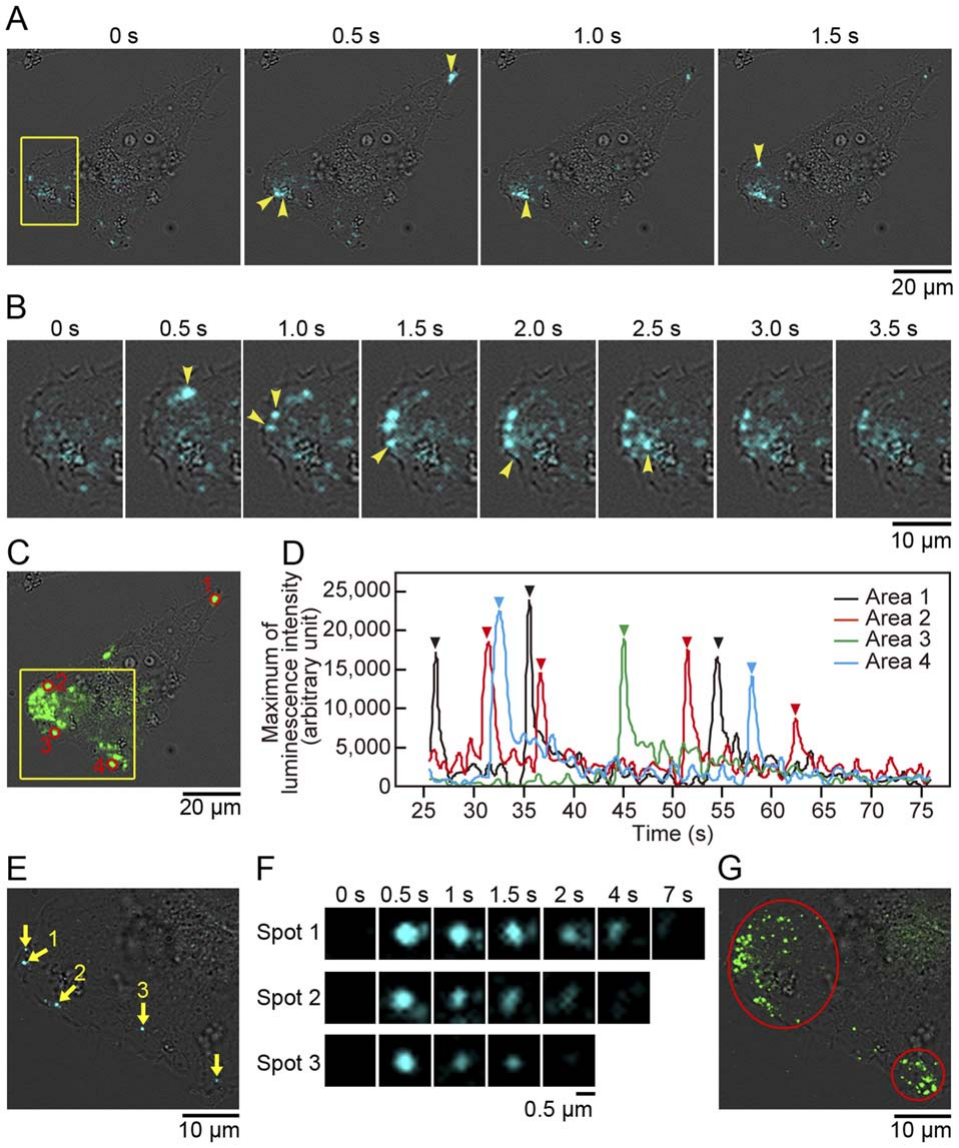
Bioluminescence images of MMP2-GLase secretion in a migrating HeLa cell. Luminescence images of MMP2-GLase secretion after the disappearing of luminescence signals of the membrane-associated MMP2-GLase. (A–D) Data of luminescence images after 25 s from the start of recording with 40× objective lens shown in Figure 3 (Video image is in Video S4). (E–G) Data of luminescence images of the leading edge obtained with 100× objective lens, after the recording with 40× objective lens (Video image is in Video S5). (A) Time-dependent luminescence signals of MMP2-GLase secretion. The first image (T = 0) is one frame in Video S4 at 35 s 54 ms after the start of recording. Luminescence spots newly appeared in a frame are indicated by yellow arrowheads. (B) Magnified luminescence images of the leading edge corresponding to the area indicated by a yellow square in (A). The first image (T = 0) is one frame in Video S4 at 51 s 79 ms after the start of recording. Newly appeared luminescence spots are indicated by yellow arrowheads. (C) An image of the maximum luminescence intensity obtained with an exposure time of 500 ms for 50 s after the disappearance of luminescence signals of MMP2-GLase. The image was made from the frame at 26 s 541 ms to the end frame at 1 min 16 s 118 ms (100 frames) after the start of recording. The luminescence signals of maximum intensity are green-colored. (D) Time-dependent changes of the maximum luminescence intensity in the area of red circles (1–4) in (C). (E) A luminescence image of MMP2-GLase on the leading edge obtained with 100× objective lens in the area of a yellow square in (C). The image is one frame in Video S5 with an exposure time of 500 ms at 43 s 567 ms after start of recording. The yellow arrows indicate luminescence signals of MMP2-GLase secretion, and luminescence spots newly appeared in this frame of the video image are numbered (spot 1–3). (F) Time-dependent change of magnified luminescence spots in (E). The magnified images of spot 1–3 at 0.5 s correspond to the spots indicated by arrow 1–3, respectively, in (E). (G) An image of the maximum luminescence intensity on the leading edge obtained with an exposure time of 500 ms for 100 s with 100× objective lens. The luminescence signals of maximum intensity are green-colored and the areas with the strong luminescence spots were red-circled.

After the disappearance of continuous luminescence signals on the cell surface of HeLa cells, the luminescence signals from exocytotic secretion of MMP2-GLase were mainly observed at the leading edge (Figure 6A and B; Video S4) and were diffused in a few seconds. This result indicated that the leading edge is the major domain to supply MMP-2 into the extracellular space of a migrating cell. Occasionally, individual MMP2-GLase secretion sequentially occurred within a few seconds along the leading edge (Figure 6B). The luminescence image of maximum luminescence intensity superimposed on the bright-field image clearly showed that the secretion sites of MMP2-GLase are close to the leading edge (Figure 6C). Further, the luminescence signal of MMP2-GLase was also observed at the trailing edge of a migrating cell (Figure 6C; Video S4). The frequency of MMP-2 secretion in each hot spot (ca. 2.5 µm in diameter) was estimated by calculating the maximum luminescence intensities of MMP2-GLase in the video image (Figure 6D). Thus, the repeated secretion of MMP-2 was observed in the specific area towards the leading and the trailing edges of the migration cell. Presumably, microtubule may define the sites for MMP-2 secretion, as previously reported [31], [32]. The secretion of MMP2-GLase was not stimulated by elevated K^+^ (data not shown), suggesting that the depolarization-independent regulatory mechanism for the sequential exocytosis of vesicles containing MMP-2 might be present.

To obtain high-resolution images of luminescence spots with the EM-CCD camera (pixel size = 16 µm), we used a 100× objective lens (NA1.45) instead of the 40× objective lens. With the 100× objective lens, each luminescence spot of MMP2-GLase secretion at the leading edge was observed more clearly (Figure 6E; Video S5) and the size of luminescence spots of MMP2-GLase secretion was as small as in 1 µm (Figure 6F). Previously, the sizes of vesicle containing MMP-2 in lammelipodia of melanoma cells were estimated to be 0.5–1 µm using the fluorescence imaging method [31]. Thus, the luminescence spots derived from secreted MMP2-GLase were interpreted as a single exocytotic vesicle. An image of maximum luminescence intensity made from successive luminescence images showed the localized sites of MMP2-GLase secretion on the leading edge with a high spatial resolution (Figure 6G). During observation for 100 s, the numbers of transient luminescence spots in the upper and lower area defined in Figure 5G were estimated to be at least 70 and 20, respectively (Video S5). Thus, the high-resolution video image of luminescence spot showed frequent vesicle fusion for MMP-2 secretion at the leading edge.

**Figure 7:**
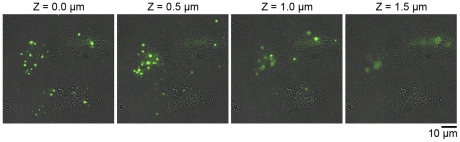
Identification of MMP2-GLase secretion from the bottom side of cell body. Bioluminescence imaging of HeLa cells transiently expressing MMP2-GLase was performed using an IX81-ZDC Zero Drift microscope system. Objective lens; 60×. Luminescence video images were obtained with an exposure time of 500 ms for 50 s after 4 min from the addition of HBSS buffer containing coelenterazine. On recording the luminescence images of MMP2-GLase secretion from cells attached on the cover slip, the in-focus z-axis positions were adjusted to 0, 0.5, 1.0, or 1.5 µm from the upside of the cover slip on a microscope stage using the Zero Drift system. Images of maximum luminescence intensity were made from all frames (100 images for 50 s) at the z-axis positions (Z) of 0, 0.5, 1.0, and 1.5 µm.

**Figure 8:**
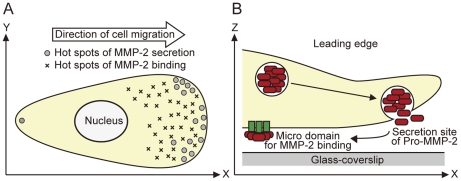
A schematic representation of MMP-2 secretion and binding on the cell surface in migration cells. (A) Hot spots of both secretion and binding of MMP-2 on the cell surface in a migrating cell. (B) Inactive pro-form of MMP-2 is frequently secreted on the leading edge and then bind to micro areas on the cell surface.

In our imaging procedure, the focus of luminescence spots for MMP2-GLase secretion was within ∼1 µm from the cover slip (Figure 7), and both luminescence spots of secreted MMP2-GLase and the membrane-associated MMP2-GLase were presumably present in the bottom side of a cell membrane (Figure 5 and Video S4). These results suggested that inactive pro-MMP-2 secreted from the under membrane at the leading edge and then bound to specific sites on the bottom side of migrating cells (Figure 8). The specific luminescence spots of MMP-2 on the cell surface might indicate the micro domains for activation of pro-MMP-2 or for binding of active MMP-2. Our video-rate imaging method using the fusion protein of MMP2 with GLase can be applied to investigate molecular mechanism for exocytotic secretion of MMP-2 in various types of cells including cancer cells. As the polarized distribution of other proteins including metalloproteinases and integrins have been investigated in migrating cells [17], [33], [34], the video-rate bioluminescence imaging might be useful to investigate the secretion and binding of these proteins in living cells.

In conclusion, we have demonstrated for the first time the visualization of the exocytotic secretion of MMP-2 from a migrating cell in real time using bioluminescence video imaging. This method showed the repeated secretion of MMP-2 from specific sites at the leading edge of the cell. The bioluminescence video imaging is a powerful approach to investigate distribution and dynamics of proteins on the whole surface of polarized cells in real time.

## Materials and Methods

### Plasmids

The secreted *Gaussia* Luciferase (GLase, K18 to D185) with the signal peptide sequence (Figure 1D) was expressed in HeLa cells (ATCC CCL-2) using pcDNA3-GLuc (Prolume Ltd., Pinetop, AZ).

To express the fused protein of hMMP-2 to GLase (MMP2-GLase), the vector pcDNA3-hMMP2-GLuc was constructed as follows. The *Hin*dIII-*Eco*RI cDNA fragment coding human MMP-2 preproprotein was obtained from IMAGE cDNA clone 3161383 by PCR amplification (25 cycles; 15 sec at 98°C, 15 sec at 55°C, 2 min at 68°C) using KOD-plus-DNA polymerase (Toyobo Co., Osaka, Japan) with a primer set of hMMP2-P1 (5′ ggc AAGCTT AGCCACC **ATG** GAG GCG CTA ATG GCC C 3′; *Hin*dIII site underlined, methionine bolded) and hMMP2-P2 (5′ ggc GAATTC GCA GCC TAG CCA GTC GGA T 3′; *Eco*RI site underlined). The PCR fragment was digested with *Hin*dIII and *Eco*RI, and then inserted into pcDNA3-GLuc-BE [26] to give pcDNA3-hMMP2-GLuc. The protein consisting of the signal peptide sequence of MMP-2, pro-MMP-2 and GLase was expressed (Figure 1D).

To express wild type human MMP-2 and FLAG (DYKDDDDK) tagged human MMP-2 (MMP2-FLAG) at the carboxyl terminus in HeLa cells, the vectors of pcDNA3-hMMP2 and pcDNA3-hMMP2-Flag were constructed as follows. For pcDNA3-hMMP2, the *Eco*RI-*Xba*I fragment of human MMP-2 cDNA was obtained from IMAGE cDNA clone 3161383 by PCR amplification (25 cycles; 15 sec at 98°C, 15 sec at 55°C, 2 min at 68°C) using KOD-plus-DNA polymerase with a primer set of hMMP2-P1 and hMMP2-P3 (5′ ccc TCTAGA **TTA** GCA GCC TAG CCA GTC GGA TTT GAT 3′; *Xba*I site underlined, stop codon bolded). The PCR fragment obtained was digested with *Hin*dIII and *Xba*I, and then was inserted into *Hin*dIII/*Xba*I sites of pcDNA3 to give pcDNA3-hMMP2. For pcDNA3-hMMP2-Flag, the *Eco*RI-Flag-TAA-*Not*I linker (GAC TAC AAA GAC GAT GAC GAC AAG for Flag sequence) was replaced with the *Eco*RI/*Not*I fragment of *GLuc* in pcDNA3-hMMP2-GLuc to give pcDNA3-hMMP2-Flag.

### Cell culture and transfection

HeLa cells were cultured in DMEM (D5796; Sigma, St. Louis, MO) supplemented with 10% fetal bovine serum (Invitrogen, Carlsbad, CA). For transient expression, HeLa cells were transfected with an expression vector using Fugene HD (Roche Applied Science, Mannheim, Germany). The transformed cells were cultured for 24 h at 37°C in a humidified 5% CO_2_ incubator.

### Measurement of GLase activity using a luminometer

HeLa cells (2×10^5^ cells) cultured in a Falcon 6 well-plate (BD Bioscience, Bedford, MA) were transfected with 2 µg of GLase-expression vectors and 6 µl of Fugene HD (Roche Applied Science), and then cultured for 24 h. After washing 3 times with 3 ml of PBS at room temperature, HeLa cells were incubated for 60 min at 37°C with 1 ml of HBSS and 5 µl of the conditioned medium was used for determining the luminescence activity of GLase by addition of 50 µl of HBSS containing coelenterazine (3 µg/ml, Chisso Co., Tokyo, Japan). The initial maximal light intensity was measured using an Atto (Tokyo, Japan) AB2200 luminometer (ver. 2.56, rev. 5.14n) equipped with a R4220P photomultiplier (Hamamatsu Photonics, K.K.).

### Western blot analysis

Western blot analyses were performed using anti-GLase rabbit polyclonal antibody (Prolume) and anti-MMP-2 mouse monoclonal antibody (F-68, Clone No. 42-5D11, Daiichi Fine Chemical Co. Ltd., Toyama, Japan) as follows. HeLa cells (2×10^5^ cells) cultured for 24 h in a Falcon 6-well plate (BD Bioscience) were transfected with 2 µg of pcDNA3-hMMP2, pcDNA3-hMMP2-Flag, pcDNA3-hMMP2-GLuc, or pcDNA3 (as a control vector) and 6 µl of Fugene HD (Roche Applied Science), and then cultured for 24 h. To analyze the secreted proteins into conditioned medium, the transfected cells were washed 3 times with HBSS and further cultured in 1 ml of HBSS for 24 h, and then the conditioned medium was 10-fold concentrated using an Ultrafree-0.5 centrifugal filter device with Biomax-10 membrane (MWCO 10 kDa, Millipore, Billerica, MA). The concentrated sample (12 µl) was added with 5× Laemmli's sample buffer for electrophoresis. To analyze the cell lysate, the cells were washed 3 times with PBS, lysed with 100 µl of 1× Laemmli's sample buffer, and sonicated for 10 s with an UP50H ultrasonic processor with 1 mm diameter tip (intensity = 0.4, Hielscher, Teltow, Germany). The proteins of the cell lysate and the conditioned medium in 15 µl of the 1× Laemmli's sample buffer were separated by SDS-PAGE (SuperSep Ace 10% or 5–20% gel, Wako, Osaka, Japan) under reducing conditions. The separated proteins were transferred electrically to a PVDF membrane (Bio-Rad, Hercules, CA) and then the membrane was incubated for 1 h with PBS containing 5% skim milk. After incubation for 1 h at room temperature with the primary anti-GLase or anti-MMP-2 antibodies (1∶1000), the membrane was further incubated for 1 h with the secondary HRP-conjugated anti-rabbit or anti-mouse IgG antibodies (1∶5000, GE Healthcare UK Ltd). The proteins on the membrane were visualized using an Immobilon Western blotting kit (Millipore) and a LAS-4000mini image analyzer (Fuji film, Tokyo, Japan).

### Immunofluorescence analysis

To determine localization of MMP2-GLase expressed in HeLa cells, immunofluorescence analysis was performed using HeLa cells co-expressed with MMP2-FLAG. HeLa cells (4×10^4^ cells) cultured for 24 h in a Falcon 4-well chamber slide (BD Bioscience) was co-transfected with pcDNA3-hMMP2-GLuc and pcDNA3-hMMP2-Flag (0.125 µg each) with 1.5 µl of Fugene HD (Roche Applied Science), and then cultured for 24 h. All procedures for immunostaining were performed at room temperature. The transfected cells were fixed with PBS containing 4% paraformaldehyde for 20 min, permeabilized for 15 min with PBS containing 0.1% Triton X-100, and then incubated for 30 min with PBS containing 1% fatty acid-free bovine serum albumin (Wako, Osaka, Japan). After incubating for 1 h with the primary anti-GLase rabbit polyclonal antibody (1∶1000) and anti-FLAG M2 mouse monoclonal antibody (1∶250, F1084, Sigma), the cells were further incubated for 45 min with anti-rabbit IgG antibody conjugated with Alexa Fluor 647 and anti-mouse IgG antibody conjugated with Alexa Fluor 488 (1∶2000, Invitrogen), and then mounted in Pro-long Gold antifade reagent (Invitrogen). Fluorescence images of Alexa Fluor 488 and Alexa Fluor 647 were acquired and processed using an Apotome microscope system (Carl Zeiss, Jena, Germany) with AxioVision4.6 software.

### Bioluminescence imaging

For live cell luminescence imaging, HeLa cells cultured on a 35 mm glass-bottom dish (Asahi Glass Co., Tokyo, Japan) were transfected with GLase expressing vectors and cultured for 24 h. To obtain the luminescence image, the cells were washed 3 times with 3 ml of PBS and then were soaked with 1 ml of Hank's balanced sodium solution (HBSS; Invitrogen) containing coelenterazine (3 µg/ml). Luminescence signals were monitored at 37°C using a model IX71 or IX81-ZDC microscopes (Olympus Co., Tokyo, Japan) equipped with a thermostat incubator (Tokai Hit, Shizuoka, Japan) and with a back-thinned EM-CCD camera (model C9100-13; 512×512 pixels, pixel size = 16 µm; Hamamatsu photonics, K.K., Hamamastu, Japan) in a dark box. In an IX81-ZDC Zero Drift microscope system, a camera lens adaptor with an IR cut filter was attached between the microscope and the EM-CCD camera. High numerical aperture (NA) objective lens of UPLFLN 40×O (NA1.30), UPLSAPO 60×O (NA1.35), and PlanApo 100×OTIRFM (NA1.45) (Olympus) were used. Data of bioluminescence signals were captured on a computer hard disk using AQUACOSMOS software version 2.6 (Hamamatsu photonics, K.K.) with acquisition mode of 1×1 binning, fast scanning, EM gain level = 255, and photon-counting level = 1 in the software. The luminescence signals were acquired with an exposure time of 30.5, 100, or 500 ms with an interval time of approximately 0.78 ms per image. Captured images were processed and calculation of luminescence signals was performed using the AQUACOSMOS software. In some cases, the maximum luminescence intensities in the successive luminescence images were used (using “MaxTrace” method in “sequential calculation” menu in the software) to generate an image, which consisted of maximum of luminescence intensities from successive video images at a time resolution of 500 ms.

The luminescence signals were converted to pseudo-colored images (cyan or green), and then superimposed on the bright-field image to show localization of luminescence signals on a cell. To analyze time-dependent changes in luminescence intensity in a video image, average and maximum of luminescence intensities in the defined areas were calculated.

## Supporting Information

Video S1 **Video-rate bioluminescence imaging of GLase secretion from a single HeLa cell with an exposure time of 500 ms.** Luminescence signals were recorded for 75 s with an exposure time of 500 ms and a reading time of 0.78 ms per image. Luminescence signals were converted to pseudo-colored images (cyan) and then superimposed on the bright-field image. Elapsed time from the start of recording is shown in the movie.
(MOV)

Video S2 **Video-rate bioluminescence imaging of GLase secretion from a single HeLa cell with an exposure time of 100 ms.** Luminescence signals were recorded for 15 s with an exposure time of 100 ms and a reading time of 0.78 ms per image.
(MOV)

Video S3 **Video-rate bioluminescence imaging of GLase secretion from a single HeLa cell with an exposure time of 30.5 ms.** Luminescence signals were recorded for 4.7 s with an exposure time of 30.5 ms and a reading time of 0.78 ms per image.
(MOV)

Video S4 **Video-rate bioluminescence imaging of MMP2-GLase being secreted and bound on the cell surface in a migrating HeLa cell with a 40× objective lens.** Luminescence signals were recorded for 75 s with an exposure time of 500 ms and a reading time of 0.78 ms per image using a 40× objective lens. Video S4 was obtained after the addition of coelenterazine for 20 s.
(MOV)

Video S5 **Video-rate bioluminescence imaging of MMP2-GLase secretion from a migrating HeLa cell with a 100× objective lens.** Luminescence signals were recorded for 100 s with an exposure time of 500 ms and a reading time of 0.78 ms per image using a 100× objective lens. After the acquisition of Video S4, Video S5 was immediately obtained.
(MOV)

## References

[1] Chen WT, Wang JY (1999). Ann N Y Acad Sci.

[2] Nabeshima K, Inoue T, Shimao Y, Okada Y, Itoh Y (2000). Cancer Res.

[3] Suetsugu S, Yamazaki D, Kurisu S, Takenawa T (2003). Dev Cell.

[4] Ogier C, Bernard A, Chollet AM, Le Diguardher T, Hanessian S (2006). Glia.

[5] VanSaun MN, Matrisian LM (2006). Birth Defects Res Part C Embryo Today.

[6] Seiki M (2002). Curr Opin Cell Biol.

[7] Egeblad M, Werb Z (2002). Nat Rev Cancer.

[8] Lafleur MA, Handsley MM, Edwards DR (2003). Expert Rev Mol Med.

[9] Overall CM, Kleifeld O (2006). Nat Rev Cancer.

[10] Candelario-Jalil E, Yang Y, Rosenberg GA (2009). Neuroscience.

[11] Tonti GA, Mannello F, Cacci E, Biagioni S (2009). Int J Dev Biol.

[12] Stetler-Stevenson WG, Krutzsch HC, Liotta LA (1989). J Biol Chem.

[13] Strongin AY, Collier I, Bannikov G, Marmer BL, Grant GA (1995). J Biol Chem.

[14] Atkinson SJ, Crabbe T, Cowell S, Ward RV, Butler MJ (1995). J Biol Chem.

[15] Brooks PC, Strömblad S, Sanders LC, von Schalscha TL, Aimes RT (1996). Cell.

[16] Deryugina EI, Ratnikov B, Monosov E, Postnova TI, DiScipio R (2001). Exp Cell Res.

[17] Hood JD, Cheresh DA (2002). Nat Rev Cancer.

[18] Oheim M, Loerke D, Chow RH, Stühmer W (1999). Philos Trans R Soc Lond B Biol Sci.

[19] Steyer JA, Almers W (2001). Nat Rev Mol Cell Biol.

[20] Axelrod D (2003). Methods Enzymol.

[21] Schmoranzer J, Kreitzer G, Simon SM (2003). J Cell Sci.

[22] Jaiswal JK, Simon SM (2007). Nat Chem Biol.

[23] Inouye S, Ohmiya Y, Toya Y, Tsuji FI (1992). Proc Natl Acad Sci U S A.

[24] Thompson EM, Adenot P, Tsuji FI, Renard JP (1995). Proc Natl Acad Sci U S A.

[25] Miesenböck G, Rothman JE (1997). Proc Natl Acad Sci U S A.

[26] Suzuki T, Usuda S, Ichinose H, Inouye S (2007). FEBS Lett.

[27] Ma Z, Chang MJ, Shah R, Adamski J, Zhao X (2004). J Biol Chem.

[28] Zhai Y, Hotary KB, Nan B, Bosch FX, Muñoz N (2005). Cancer Res.

[29] Verhaegen M, Christopoulos TK (2002). Anal Chem.

[30] Tannous BA, Kim DE, Fernandez JL, Weissleder R, Breakefield XO (2005). Mol Ther.

[31] Schnaeker EM, Ossig R, Ludwig T, Dreier R, Oberleithner H (2004). Cancer Res.

[32] Sbai O, Ferhat L, Bernard A, Gueye Y, Ould-Yahoui A (2008). Mol Cell Neurosci.

[33] Bretscher MS (1996). Cell.

[34] Jones MC, Caswell PT, Norman JC (2006). Curr Opin Cell Biol.

